# What does a dollar mean to you? utilizing intrinsic rewards within contingency management

**DOI:** 10.3389/fpsyt.2025.1420763

**Published:** 2025-03-21

**Authors:** Anaheed Shirazi, Joseph A. Carley, Dara G. Ghahremani, Arthur L. Brody, Ariel J. Lang

**Affiliations:** ^1^ Department of Psychiatry, University of California, San Diego, San Diego, CA, United States; ^2^ Department of Psychiatry, Veterans Affairs (VA) San Diego Healthcare System, San Diego, CA, United States; ^3^ Veterans Affairs (VA) San Diego Center of Excellence for Stress and Mental Health, San Diego, CA, United States; ^4^ Department of Psychiatry and Biobehavioral Sciences, University of California, Los Angeles, Los Angeles, CA, United States

**Keywords:** contingency management (CM), integrative treatment approaches, substance use disorder, addiction, intrinsic reward, Integrative Contingency Management

## Abstract

Substance Use Disorders (SUDs) pose a significant public health challenge. Medications used for treatment of SUDs are limited in their efficacy, particularly in cannabis and stimulant use disorders, and non-pharmacological interventions have shown, at most, moderate effectiveness, leaving a continuing need for improved treatments. Contingency management (CM) is an evidence-based treatment with promising results, operationalized in SUD treatment programs by using monetary rewards to help patients achieve target behaviors such as abstinence. Several factors limit the viability of CM as a sustainable, effective treatment, suggesting insufficiency of monetary reinforcement alone. Implementation can be costly, requiring increased monetary reinforcers over time to reach target behaviors, and effects do not endure. We propose an integrative model of CM that explicitly incorporates intrinsic rewards into CM to enhance its effectiveness. This model involves redirecting salience attribution of monetary rewards towards goals and activities that are personally relevant and motivating for the individual that do not involve substance use. This integrative model of CM may address current challenges of and some of the barriers to implementation of CM in clinical practice.

## Highlights

- While internal motivation plays an important role in substance use recovery, it remains underexplored in Contingency Management.
- We hypothesize that merging intrinsic and extrinsic incentives to develop an integrative model of CM may improve its effectiveness.
- We hypothesize that remodeling of salience attribution is an underlying mechanism for the improved effectiveness of Integrative CM.

## Introduction

### Current state of substance use disorders treatments

Substance use disorders (SUDs) are complex psychiatric conditions with genetically and environmentally influenced biological and behavioral components (1). Therefore, recovery and rehabilitation programs designed to treat SUDs often leverage a multimodal approach that includes both pharmacological and non-pharmacological interventions (1). The number of medications that are FDA approved to treat substance use disorders and their efficacy remain limited, particularly for stimulant use disorder and cannabis use disorder, and in the adolescent populations (2–5). Cognitive, behavioral, and motivation/reward-based strategies have been shown to be at best moderately effective in short-term treatment of substance use disorders (6).

With the fourth wave of drug overdoses on the rise in the U.S., it is crucial to expand and enhance treatment strategies to address this growing crisis. Stimulants are playing a major and increasing role in this crisis (7). While no FDA-approved medications currently exist for stimulant use disorder, a behavioral intervention, contingency management (CM), has demonstrated robust efficacy. It is crucial to consider novel strategies that may address implementation barriers and improve the effectiveness of existing interventions, especially to improve adherence and long-term outcomes (8).

### Contingency management: a promising, underutilized non-pharmacological approach

CM is an evidence-based treatment operationalized in SUD treatment programs by using monetary rewards to help patients achieve target behaviors of abstinence from substance use and attendance to treatment sessions (9). CM is one of the most promising non-pharmacological approaches for treatment of SUDs, and its effectiveness has been well-established (10). Systematic review and meta-analysis studies provide evidence supporting the use of CM in the treatment of SUDs (11–13). Results of a randomized controlled trial comparing CBT and CM for stimulant use disorders suggested that CM is superior to CBT, with better retention and lower use of stimulants during study participation (14).

Despite evidence indicating the success of CM in SUD recovery efforts, several limitations contribute to its lack of widespread availability. These include the requirement of large financial resources to supply monetary rewards and resistance among practitioners to incentivize patients using monetary rewards. With respect to the former, CM has primarily been delivered using two major protocols that differ based on whether rewards are delivered via a voucher or a randomly-selected prize from a “fishbowl” (15). The magnitude and immediacy of reward are factors that contribute to effective CM (15). Increasing the magnitude of reward has been shown to improve measures of abstinence (16, 17). However, increasing monetary reinforcers indefinitely to increase effectiveness of treatment is often too costly, and the amount of monetary resources required to expand CM programs and improve outcomes is a limitation. Additionally, diminished enthusiasm from counsellors and clinicians towards incentivizing abstinence with tangible rewards such as monetary prizes has been identified as a barrier to the clinical implementation of CM by prior studies (18, 19). A 12-step treatment ideology that emphasizes self-reflection, personal responsibility, commitment to ongoing self-improvement and helping others, was shown to be negatively associated with acceptance of the CM concept (18, 19). Also, some have argued that reliance on extrinsic rewards may take attention away from internal motivation (10) for recovery. For example, in a developmental context, children have been found to lose their enthusiasm for an enjoyable activity after they are explicitly rewarded for it, highlighting the potential “detrimental effects” of extrinsic rewards on intrinsic motivation (20, 21). This is while others have suggested that reward contingencies do not affect intrinsic motivation negatively (22). Meta-analyses by Cerasoli et al. suggest that incentives and intrinsic motivation are not necessarily antagonistic and may have complementary roles (23).

Although the use of intrinsic rewards within the CM context may reorient patients towards this internal motivation and enhance the effects of CM rewards, it remains underexplored. One study showed that motivational interviewing (supporting intrinsic rewards) has greater long-term benefits than CM (extrinsic rewards) (24), consistent with results of other studies suggesting that CM loses its effectiveness over time (25). Such results highlight the importance of coupling CM with interventions that will empower individuals to tap into their intrinsic motivation to continue sobriety after the CM rewards are removed.

CM efficacy has been mainly explained by principles of operant conditioning: the theory of learning where behaviors are influenced by their consequences (negative and positive reinforcers). Within this framework, behaviors that are positively reinforced/rewarded are more likely to be repeated (e.g., abstinence or treatment adherence) (26). Another hypothesis proposed to explain the mechanisms through which CM exerts its benefits involves deliberative decision-making processes. Presenting concrete and immediate rewards provided by CM engages deliberative processes, which in turn improves the ability of these processes to attend to non-drug options (27). This may have similarities to therapeutic mechanisms of mindfulness-based interventions in addiction, which involve breaking the automaticity of substance use and improving awareness over cognitive and behavioral processes (28). A missing aspect in existing CM approaches is accounting for participants’ intentions and motivations for earning monetary rewards.

Previous studies that used cash rewards for performance improvement in the workforce offer insight as to the importance of context under which external rewards, such as monetary rewards, are presented. Landry et al. showed that external rewards can be leveraged to enhance individuals’ performance when they are presented in a way that positively contributes to their psychological needs and elicits intrinsic motivation (29). The definition of intrinsic and extrinsic rewards and motivation varies across contexts. Some consider an act intrinsically motivating if a person engages in the activity for its inherent satisfaction rather than a separable consequence, while extrinsic motivation involves performing an activity to achieve an external outcome. In operant conditioning theory, all behaviors are motivated by separable consequences, such as food or money, and intrinsically motivated activities are said to be those for which the reward is entailed in the activity itself (30).

In current CM approaches, the emphasis is on reinforcing abstinence by using external rewards (money, vouchers, etc.). There is also growing evidence indicating the effectiveness of interventions that provide alternative sources of reinforcement by restructuring the environment, such as the community-reinforcement approach (31), behavioral activation, and substance-free activity sessions (32, 33).

In this paper, we hypothesize that a potential way to enhance CM effectiveness and adherence is by leveraging multiple motivational pathways and emphasizing a focus on making the act of abstinence more rewarding in itself, driven by intrinsic motivations. Neuroimaging studies show that neural activity patterns during the decision-making process about task engagement differ depending on whether the motivation is intrinsic (fun, enjoyment, interest) or extrinsic (money, reward, incentive, prize) (34). Such results highlight the potential for additive effects in an integrative approach. Moreover, other neuroimaging studies suggest that those with substance use disorders show limited self-awareness linked to deficits in ventromedial PFC function and indicate that interventions targeting personal relevance may have a significant impact on therapeutic outcomes (35).

We propose an integrative version of CM, in which intrinsic rewards are achieved by attaching personal meaning and values to the external rewards.

### The hypothesis: an integrative model of CM

We hypothesize that the integration of intrinsic and extrinsic rewards within CM will increase the effectiveness of the intervention while addressing the above-mentioned limitations of current CM approaches. Specifically, integrating personal motivations and goals that are based on individuals’ intrinsic values (what we define as “values-based intrinsic rewards”, such as “spending quality time with family” or “demonstrably improving physical health”) with extrinsic monetary rewards within CM will result in greater overall subjective reward value relative to monetary value alone (which may even be implicitly associated with future drug use). In this model, extrinsic and intrinsic rewards will be weighed against substance use and its consequences. Therefore, the effectiveness of the intervention would potentially improve without increasing the amount of monetary reward. Interconnected with personal values, internal motivation is thought to be a major contributor to recovery (36, 37) and is the basis of some psychotherapy modalities and recovery programs for the treatment of SUDs (37–39). Clarification and exploration of values within the work of psychotherapy enhances awareness of behaviors that are misaligned with internal values and inspire change to maintain integrity of the personal value system (40). Furthermore, explicit value clarification is an approach that aids patients in decision-making processes (41). Despite the potential of intrinsic values and rewards to guide recovery, they remain untapped in CM.

### Implementation of the integrative model of CM for substance use disorder treatment

**Figure 1:**
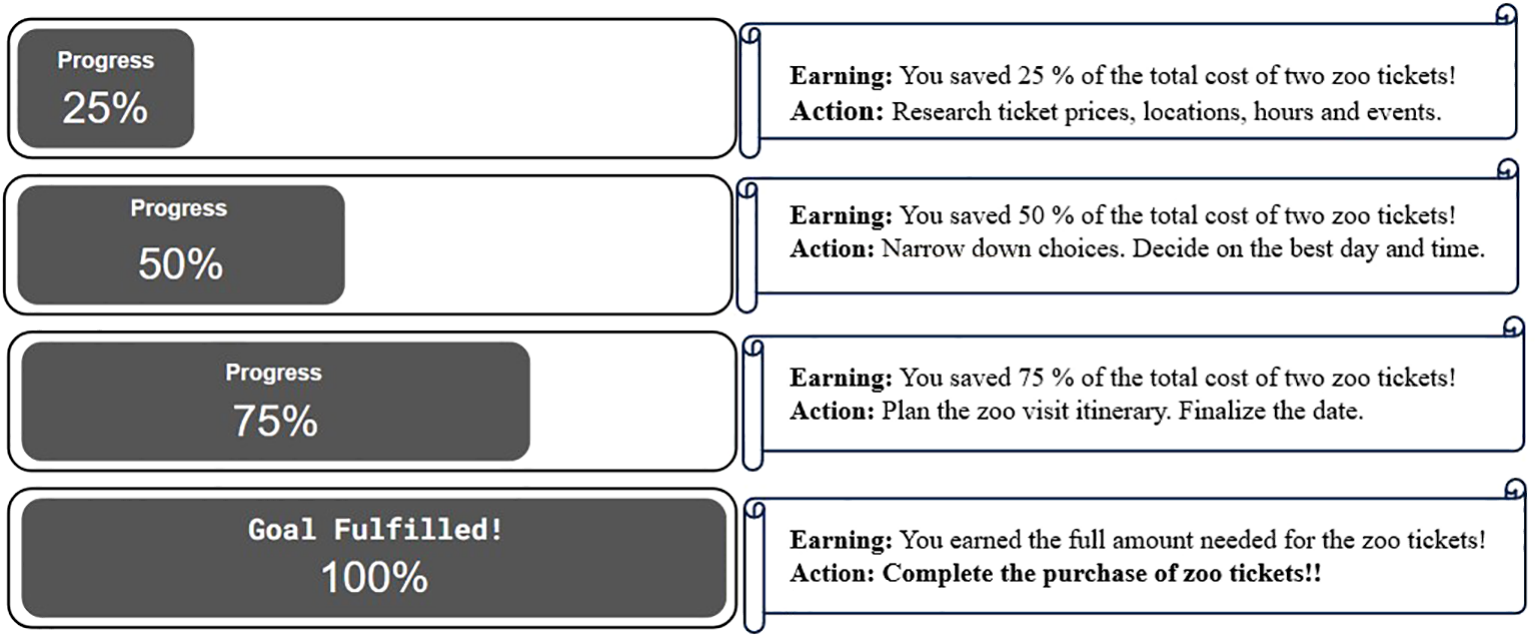
In values-based Integrative CM with independent spending models, monetary reward could be dispensed in each session along with the progress report as participants progress towards values-based collaboratively established goals. Alternatively, the amount of monetary reward could be listed in the progress report and only presented when the goal is fulfilled.

**Figure 2:**
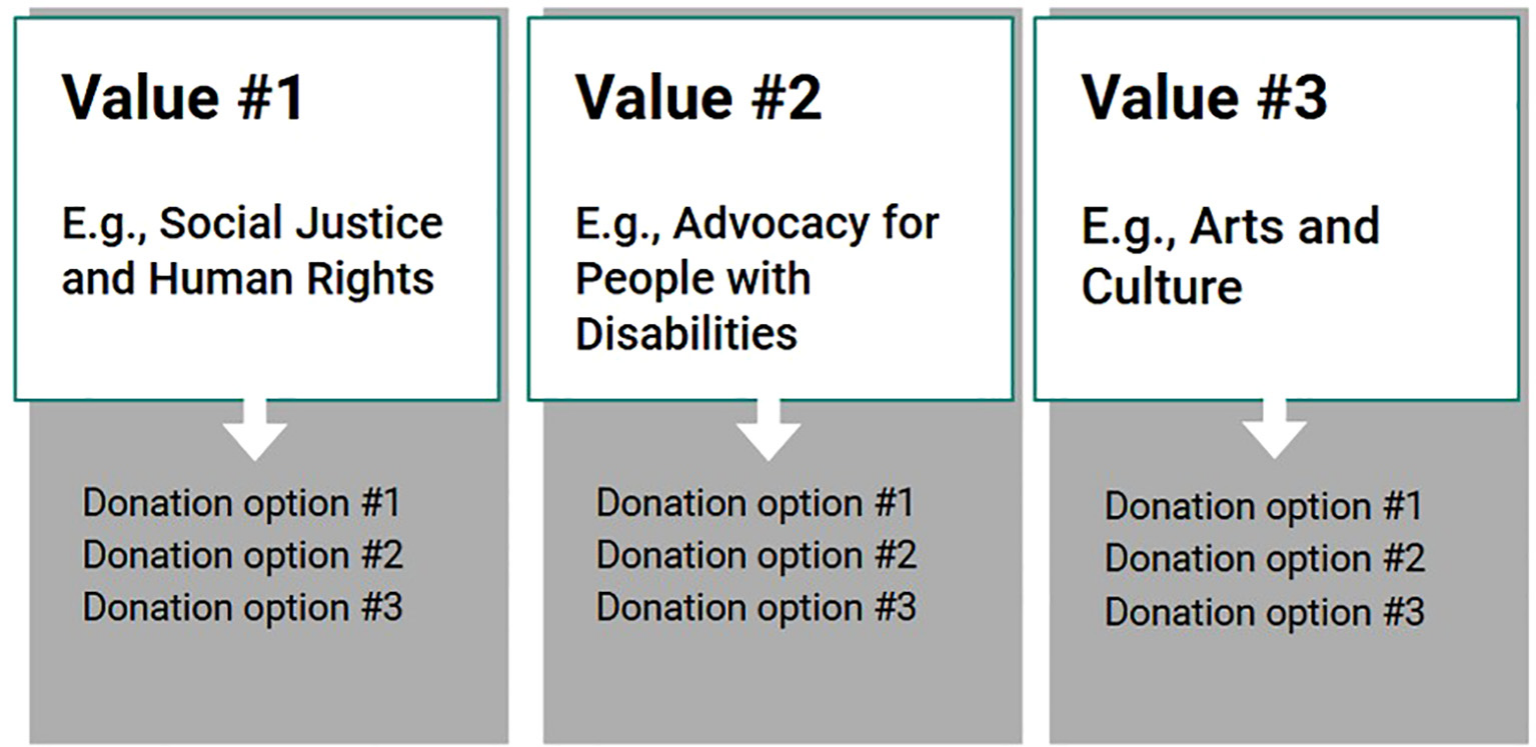
In the donation-based model of Integrative CM, participants could choose from several donation options built into the model based on their personal values.

**Figure 3:**
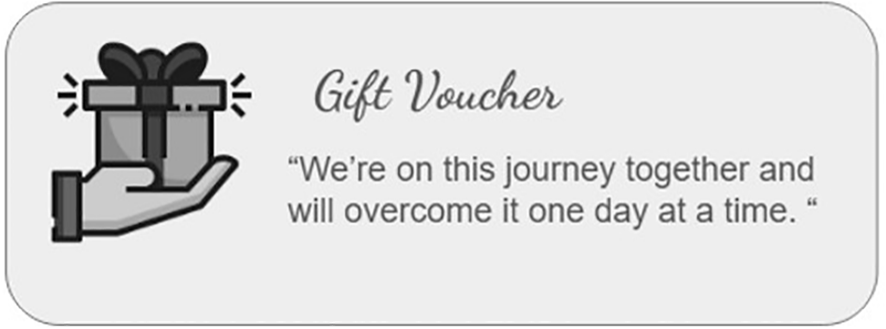
In the collaborative model of Integrative CM, the participants may gift their earned external reward to another participant, and add an encouraging note to foster a sense of community and support.

Implementation of Integrative CM may begin with a values-exploration and clarification session (adapted from Acceptance and Commitment Therapy) (42, 43) in which the CM reward is collaboratively-established based on the individual’s personal values. As an example, if a patient values spending quality time with their grandchildren as the main motivation for recovery, the values-based CM reward could potentially be tickets to the zoo as a way to spend time with them. In subsequent sessions, the patient’s rewards would focus on progress toward zoo tickets (**Figure 1**). Another possibility would be to include charitable donation options in CM sessions, an approach previously explored for habit formation (44). For example, if a patient cares about helping the homeless population, they might choose to donate their CM reward to buy meals for the homeless (**Figure 2**). Additionally, a collaborative version could be considered in which the CM program participants would gift their earned vouchers/rewards to another participant, anonymously through the program, perhaps along with an encouraging note, while confidentiality of both participants is protected (**Figure 3**). We hypothesize that individuals with more pro-social tendencies may respond better to the collaborative and donation-based versions of Integrative CM.

Integration of intrinsic and extrinsic rewards in these models will allow for quantitative adjustments of the overall reward by increasing or decreasing the extrinsic/monetary reward. Furthermore, we suggest considering and discussing the monetary cost of the values-based rewards when establishing a goal to ensure it fits within the limits of CM external rewards.

While these models could be implemented via providers and through structured programs (e.g., using rewards to make donations, purchase zoo tickets, gym membership or gifts for family members through Integrative CM programs), setting the intention alone for how the monetary rewards will be used may have therapeutic potential. In this modification, participants would set their intention to earn the monetary rewards collaboratively with the CM provider and the patients would spend the monetary rewards independently. These models will need to be researched and optimized for enhanced acceptability, feasibility, and effectiveness. Additionally, the cost-benefit of Integrative CM should be thoroughly analyzed and compared with standard CM. This analysis should also consider how Integrative CM might benefit comorbidities and enhance participants’ overall health. For instance, if participants improve their physical activity by attending fitness classes as part of Integrative CM, the benefits could extend beyond substance use treatment, and these broader impacts should be factored into the cost-benefit discussions. With technological advances, automation and digitization of certain aspects of Integrative CM implementation should be considered once the optimal model has been identified, as these innovations could lower implementation costs while maintaining or enhancing efficiency.

The Integrative CM approach might *attach* sp*ecific personally-relevant meaning and emotions to the dollar value and potentially increase its significance and saliency* and as such, may enhance the likelihood of *engaging deliberative or goal-directed processes.* The value of the non-drug option (monetary reward) in CM will therefore partly be determined by specific personal values associated with CM, as clarified during initial therapeutic sessions.

## Discussion

### Potential mechanisms: remodeling of salience attribution

**Figure 4:**
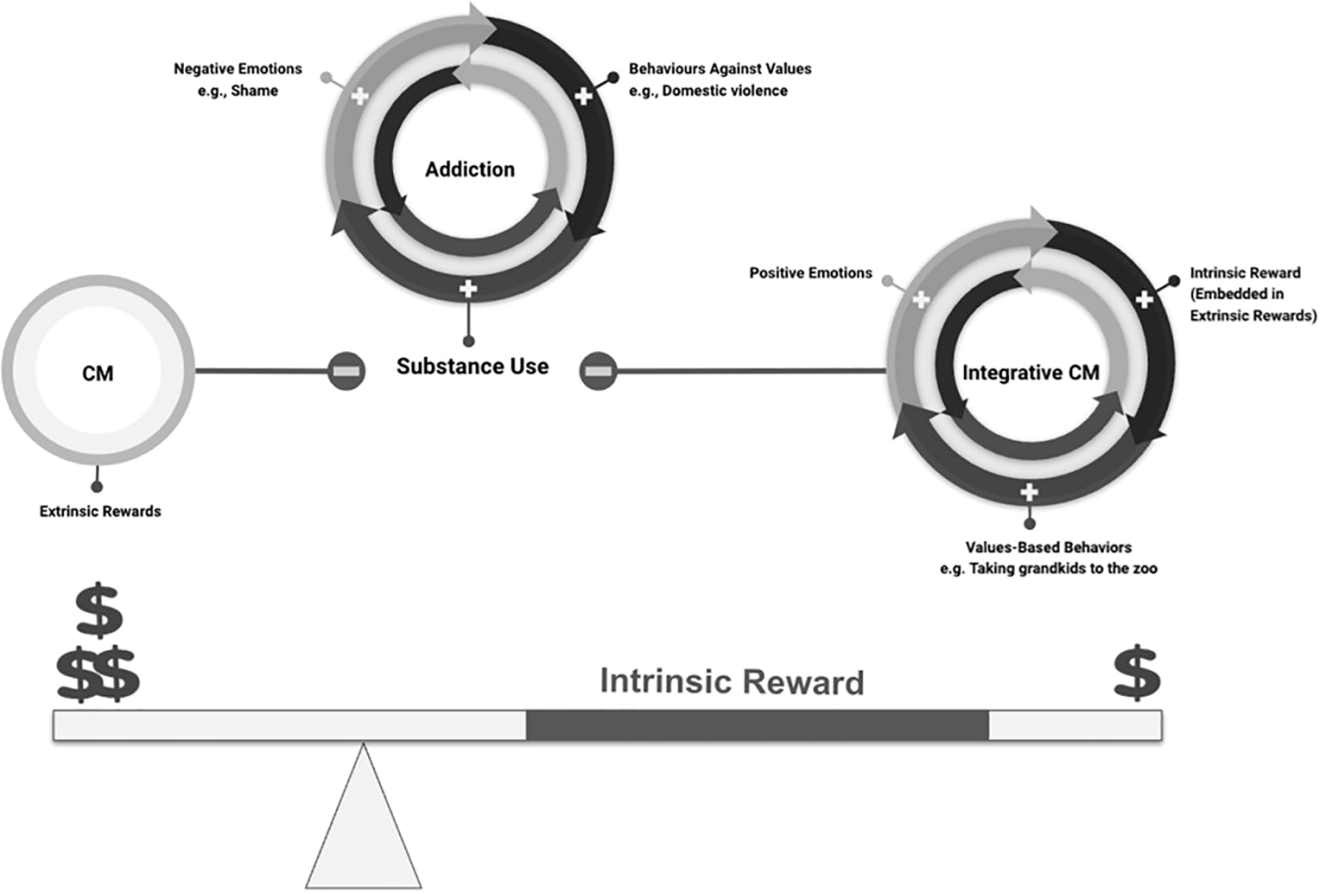
Integrating intrinsic and values-based rewards into CM enhances the magnitude of the perceived monetary reward.

Addiction-related decision making has been suggested to arise from misattribution of salience to drug-related stimuli, and attentional bias for drug-related stimuli presumably driven by reshaping of dopaminergic neural networks during the progression of substance use (45–47). The integrative model of CM may improve decision-making outcomes by *enhancing saliency of non-drug options* (monetary reward) and *attentional bias modification* (see **Figure 4**), as a result of *bringing personal meaning and value to the monetary reward*. Furthermore, monetary reward in CM may function as a cue for future drug use (48), whereas in Integrative CM, the association between money and future drug use may be modified, with money being associated with personally meaningful goals. Such an approach may mitigate concerns likening contingency management (CM) to “bribery” or deeming it “unethical” and gain broader acceptance within society compared to simply offering monetary rewards (49).

Integrative CM may also have the benefit of *encouraging participants to consider others (vs only self) in the decision-making processes* when offering prosocial rewards. In the examples of “zoo tickets” and “donation to the homeless”, the patient would be required to think about the impact of their decisions and choices (substance vs. substance-free) on others, which may allow for introspection and engagement in reasoning about the choice to consume drugs or not given their established personal goals. This pro-social orientation has been shown to impact therapeutic outcomes. Prior research demonstrates that in Narcotics Anonymous/Alcoholics Anonymous being a sponsor and providing direction and support to peers with SUD was associated with longer abstinence (50). In another study, the addition of social reinforcement to an aftercare substance recovery program was associated with increased adherence to treatment sessions (51). Furthermore, neuroimaging studies show that donating money activates similar reward-related centers in the brain as receiving money, with anterior parts of the prefrontal cortex distinctly activated during altruistic choices (donation), suggesting that engaging in donations could potentially tap into new therapeutic pathways during CM treatment (52). Even when transfers to a charity are mandatory (tax-like), neural activity in areas linked to reward processing is elicited (53).

Patients with SUD may have lost opportunities to preserve their values, contribute to their family and society, and achieve goals that they value due to their substance use. Prior research has suggested purpose in life as a promising target for preventive and intervention efforts in addressing substance use (54, 55). In Kim et al’s study, people with the highest quartile of purpose had a significantly lower likelihood of future drug misuse compared to those in the lowest quartile (54). In another study, greater pre-treatment purpose in life was a significant predictor of better response to a 30-day residential substance use treatment program among individuals with cocaine dependence (55). We hypothesize that Integrative CM will allow patients to revisit their values and potentially *experience values-based positive emotions during recovery*. Making choices that reflect their values within CM may translate into enhanced lifestyle choices, aligned with their personal values, outside of the treatment setting. This may, in turn, contribute to shifting patterns of behavior after recovery and increase the durability of CM’s effects. (see **Figure 4**). Therefore, Integrative CM could help address implementation challenges by mitigating concerns that external reinforcers may not produce lasting benefits beyond the duration of the intervention (49).

While higher incentive amounts have been linked to improved outcomes in CM (56), prior research suggests that participants’ income may not necessarily impact CM effectiveness (57, 58). However, it is also important to note the limitations of prior studies. CM research has generally focused on populations with low socioeconomic status, and the value of a given monetary amount may differ within and across individuals with varying socioeconomic status (57). The integrative approach of CM, with its emphasis on personalization, may account for varying socioeconomic status by tailoring incentives to align individual values and motivations.

As has been described, by tailoring the CM process to patients’ personal values, the integrative model of CM *is sensitive to individual differences and offers a personalized treatment.* The proposed treatment model brings focus to one’s own sense of purpose and meaning in life within the context of CM.

We hypothesize that a dollar coupled with intrinsic reward and opportunities to engage in substance-free activities has higher salience and is a stronger reinforcer than a dollar without (**Figure 4**). As per operant conditioning learning theory, a conditioned response (substance use) may be weakened by increasing reinforcement of an alternative behavior (59). Similarly, the theory of value-based decision-making in recovery posed by Field et al. suggests that effectiveness of CM may be mediated by an augmentation of cumulative subjective value for non-substance alternatives along with suppression of cumulative subjective value for substance use that facilitates a shift in reinforcer preferences and rebalances the relative value of substance use versus substance free behavior (60). In the Integrative CM model, the augmentation of evidence accumulation for substance free activities would be even further enhanced as, in addition to the monetary rewards, the model *provides opportunities to engage in alternative sources of substance-free reinforcement within the CM context*. This claim is also supported by growing evidence indicating effectiveness of interventions that provide alternative sources of reinforcement by restructuring the environment, such as the community-reinforcement approach (31), behavioral activation and substance-free activity sessions (32, 33).

### Preclinical studies that support the hypothesis

The majority of animal studies focus on reinforcement models involving primary extrinsic rewards (e.g., food, drugs), but a few pre-clinical studies that examine choice between such rewards and socially-oriented rewards lend support for our hypothesis. Venniro et al. (2021) showed the protective effects of social interaction in rat models of cocaine addiction. Rats’ cocaine self-administration was significantly decreased when they were presented with a lever that would give them access to a social peer. All rats chose social interaction over substance use on all occasions except for when the social reinforcement was significantly delayed or associated with a punishment (61). Not only does this study illustrate the potency of social rewards, but it aligns with the idea supported by prior research that reward from one source can replace or substitute for reward from another. In another study of Prairie voles, social bonding was shown to decrease choices for amphetamine reward in this monogamous mammalian species (62). In humans, however, these models can become complicated and vary from person to person depending on individuals’ values and preferences (61), highlighting the importance of employing a personalized treatment approach.

## Future directions

We suggest potential clinical utility in merging intrinsic and extrinsic incentives to develop an integrative model of CM. However, our account is hypothetical, and we encourage future research to assess acceptability and feasibility of such a model, and to compare its effectiveness and durability of effects for producing favorable clinical outcomes (adherence to treatment, achieving abstinence, relapse prevention) relative to current CM approaches in clinical trials. Furthermore, we have introduced a few versions for Integrative CM and encourage researchers to assess and optimize these models in future studies.

## Data Availability

The original contributions presented in the study are included in the article/supplementary material. Further inquiries can be directed to the corresponding author.
